# The Impact of Systolic Blood Pressure, Pulse Pressure, and Their Variability on Diabetes Retinopathy among Patients with Type 2 Diabetes

**DOI:** 10.1155/2022/7876786

**Published:** 2022-03-22

**Authors:** Qingqing Lou, Xue Chen, Kun Wang, Huanhuan Liu, Zongjun Zhang, Yaujiunn Lee

**Affiliations:** ^1^Department of Endocrinology, The First Affiliated Hospital of Hainan Medical University, Haikou, 570102 Hainan, China; ^2^Jiangsu College of Nursing, Huaian, 223023 Jiangsu, China; ^3^Department of Endocrinology, Hainan General Hospital, Haikou, 570311 Hainan, China; ^4^Radiology Department, Affiliated Hospital of Integrated Traditional Chinese and Western Medicine, Nanjing University of Chinese Medicine, Nanjing, 210028, Jiangsu Province, China; ^5^Lee's Clinic, No. 396, Guangdong RD, Pingtung City, Pingtung County, 900, Taiwan

## Abstract

**Objectives:**

To evaluate the effects of variations in systolic blood pressure (SBP) and pulse pressure (PP) on diabetic retinopathy (DR) in patients with type 2 diabetes.

**Methods:**

A total of 3275 type 2 diabetes patients without DR at Taiwan Lee's United Clinic from 2002 to 2014 were enrolled in the study. The average age of the patients was 65.5 (±12.2) years, and the follow-up period ranged from 3 to 10 years. Blood pressure variability was defined as the standard deviation (SD) of the average blood pressure values over the entire study period and was calculated for each patient. The mean SD for SBP was 11.16, and a SBP ≥ 130 mmHg (1 mmHg = 0.133 kPa) was defined as high SBP. Based on these data, patients were divided into four groups as follows: group 1 (G1, mean SBP < 130 mmHg, SD of SBP < 11.16 mmHg), group 2 (G2, mean SBP < 130 mmHg, SD ≥ 11.16 mmHg), group 3 (G3, mean SBP ≥ 130 mmHg, SD of SBP < 11.16 mmHg), and group 4 (G4, mean SBP ≥ 130 mmHg, SD ≥ 11.16 mmHg). Based on a mean PP of 80 mmHg with a pulse pressure SD of 6.53 mmHg, the patients were regrouped into four groups designated G1′-G4′.

**Results:**

After adjusting for patient age, sex, and disease course, Cox regression showed that the mean and SD of SBP, pulse pressure, and their SDs were risk factors for DR. After stratifying the patients based on the mean and SD of the SBP, we found that the patients in the G4 group had the highest risk of DR (hazard ratio (HR) = 1.980, 95% CI: 1.716~2.285, *P* < 0.01) and patients in the G1 group had the lowest risk. Patients in the G3 group (HR = 1.409, 95% CI: 1.284~1.546, *P* < 0.01) had a higher risk of DR compared to those in the G2 group (HR = 1.353, 95% CI: 1.116~1.640, *P* < 0.01). After the restratification of patients based on the mean and SD of the pulse pressures, it was found that patients in the G2′ group had the highest risk of DR (HR = 2.086, 95% CI: 1.641~2.652, *P* < 0.01), whilst patients in the G1′ group had the lowest risk. Also, the risk of DR in the G4′ group (HR = 1.507, 95% CI: 1.135~2.000, *P* < 0.01) was higher than that in the G3′ group (HR = 1.289, 95% CI: 1.181~1.408, *P* < 0.01).

**Conclusions:**

Variability in SBP and PP are risk factors for DR in patients with type 2 diabetes. The variability of PP was better able to predict the occurrence of DR than mean pulse pressure.

## 1. Introduction

Blood pressure variability (BPV), also known as blood pressure fluctuation, refers to the degree of fluctuation in blood pressure within a certain time. Quantification of BPV usually uses the SD of blood pressure readings measured over a certain time to indicate the degree of overall changes in blood pressure during that period. BPV is often independent of the average blood pressure levels and is closely related to cardiovascular and cerebrovascular damage in diabetes patients where high variability indicates cardiovascular and cerebrovascular damage [1, 2].

Diabetic retinopathy (DR) is a common microvascular complication and an important cause of vision damage and blindness in diabetes patients [3–5]. Hypertension, mainly high SBP [6, 7] and high PP [8], is a recognized risk factor for DR; however, recent studies have shown that BPV is also associated with DR in diabetes patients [9, 10]. Hata et al. [11] conducted a multicenter study in Europe showing that systolic BPV is an independent risk factor for DR in patients with type 2 diabetes mellitus (T2DM). However, in Asian patients with T2DM, the relationship between SBP, PP variability, and DR remains unclear. In this study, we aimed to evaluate the impact and variability of SBP and PP on DR in patients with T2DM.

## 2. Materials and Methods

### 2.1. Subjects

This was a prospective cohort study. Diabetes patients who visited Taiwan Lee's United Clinics from 1 January 2002 to 30 December 2014 were enrolled in the study. Patients were followed up for 3 to 10 years, with patients requested to have four follow-up visits per year. If a patient did not come for the visit as scheduled, the health care professionals would remind the patient to come to the clinics. Patients with a follow-up period less than 3 years (*n* = 557), those with blood pressure measurements taken fewer than three times per year (*n* = 402), those with missing or incomplete data (*n* = 1378), and those who did not have T2DM (*n* = 428) were excluded from the study. Finally, 3275 patients without DR at baseline were included in the study. This study was approved by the Ethics Committee of the Affiliated Hospital of Integrated Traditional Chinese and Western Medicine of Nanjing University and the Taiwan Lee's United Clinic, China (19-053-B). All participants recruited to the study provided written informed consent.

### 2.2. Data Collection

Data were collected from the database including the following: (1) demographic and clinical data, including age, sex, diabetes course, smoking, drinking, exercise habits, medication (hypoglycemic drugs, insulin, lipid-lowering drug, hypoglycemic drugs, and hypotensive drugs), height, weight, waist circumference, hip circumference, body mass index (BMI), waist-to-hip ratio, systolic pressure, diastolic pressure, and pulse pressure, and (2) laboratory data, including fasting blood that was collected every quarter (after fasting overnight for more than 8 hours). High-performance liquid chromatography (DCCT-aligned) was used to measure glycated hemoglobin A1c (HbA1c). A Roche Cobas600 automatic biochemical analyzer was used to measure the levels of total cholesterol (TC), triglyceride (TG), high-density lipoprotein cholesterol (HDL-C), and low-density lipoprotein cholesterol (LDL-C). (3) Direct ophthalmoscopy after mydriasis was performed by an ophthalmologist. The presence of microaneurysm, cotton wool spots, intracavitary microvascular abnormalities, bleeding, hard exudate, venous aneurysm, or new retinal blood vessels was defined as DR [12]. During the follow-up period, the same procedure was conducted annually.

### 2.3. Evaluation of Mean Blood Pressure and BPV

Blood pressure values with a systolic pressure in the range of 50-300 mmHg (1 mmHg = 0.133 kPa), diastolic pressure in the range of 30-180 mmHg, and PP in the range of 20-120 mmHg were recognized as effective measurement values [9]. The mean value of multiple blood pressure measurements taken from the same patient on the same day was taken as the measurement value for that day. Mean arterial pressure (MAP) was calculated as (SBP + 2 × diastolic blood pressure)/3SD. The BPVs were defined as SD from the average blood pressure [13] by calculating average values of systolic, diastolic, and pulse pressure and MAP measurements and their SDs for each participant during the entire study period.

### 2.4. Patient Grouping

A mean SD of SBP of 11.16, and a SBP ≥ 130 mmHg (1 mmHg = 0.133 kPa) were defined as high SBP. Based on these data, all the patients (*n* = 3275) were divided into four groups as follows: G1 (mean SBP < 130 mmHg, SD of SBP < 11.16 mmHg), G2 (mean SBP < 130 mmHg, SD ≥ 11.16 mmHg), G3 (mean SBP ≥ 130 mmHg, SD of SBP < 11.16 mmHg), and G4 (mean SBP ≥ 130 mmHg, SD ≥ 11.16 mmHg). Based on a mean PP of 80 mmHg and a SD of the PP of 6.53 mmHg, the 3275 patients were regrouped into four groups designated G1′-G4′: G1′ (mean PP < 80 mmHg, SD of PP < 6.53 mmHg), G2′ (mean PP < 80 mmHg, SD ≥ 6.53 mmHg), G3′ (mean PP ≥ 80 mmHg, SD of PP < 6.53 mmHg), and G4′ (mean PP ≥ 130 mmHg, SD of PP ≥ 6.53 mmHg).

### 2.5. Statistical Analysis

SPSS 22.0 software was used for statistical analysis. Count data were expressed as the number of cases (%), and a *χ*^2^ test was used for comparisons between the groups. When the measurement data were normally distributed, the data were expressed as mean ± SD, and an independent sample *t*-test was used to compare the difference between two groups. Nonnormally distributed data were expressed as medians (upper and lower quartile), and a nonparametric test was used for intergroup comparisons. Cox regression analysis was used to analyze the relationship between different blood pressure variables and the development of DR. As we grouped patients into G1-G4 according to their SPB and SD of SBP and G1′-G4′ according to their PP and SD of PP to compare between the different groups, G1 and G1′ served as the reference in the COX regression models. Covariates including age, sex, course of the disease, BMI, waist-to-hip ratio, HbA1c, TC, TG, LDL-C, and HDL-C were entered into the models simultaneously. Hazard ratios (HR) and their respective 95% confidence intervals (CI) were calculated. *P* values of <0.05 were considered statistically significant.

## 3. Results

### 3.1. Baseline Characteristics

A total of 3275 patients participated in this study. Based on the results of the fundus mydriasis test, patients were divided into a non-DR (NDR) group (2833 cases) and a DR group (442 cases) in which 100 cases had nonproliferative DR and 342 cases had proliferative DR. Among the patients with proliferative DR, 247 cases were at the early stage of proliferation, 72 cases were at the fibrotic stage, and 23 cases were at the late stage of proliferation. During follow-up, progression of DR was observed, with 15 patients progressing from nonproliferative to proliferative DR and being in the early stage of proliferation. Thirteen cases progressed from the early proliferative stage to the fibrotic stage, and three patients progressed from the fibrotic stage to the late stage of proliferation. There were no significant differences in sex, BMI, waist-to-hip ratio, smoking history, drinking history, use of lipid-lowering drugs, use of antihypertensive drugs, diastolic blood pressure, MAP, TC, TG, and LDL-C between the NDR and the DR groups (*P* > 0.05). Compared to the NDR group, patients in the DR group were older, had had a longer duration of diabetes, and had poorer exercise habits. They also had a higher incidence of macular edema and cataracts, lower use of hypoglycemic drugs and insulin, and higher levels of HbA1c, SBP, and pulse pressure. The NDR patients also had lower levels of HDL-C. The differences between the groups were all statistically significant (*P* < 0.05, Table 1).

### 3.2. The Relationship between Blood Pressure and the Risk of DR

After adjusting for age, sex, and disease course, Cox regression showed that a higher mean SBP (hazard ratio (HR) = 1.023, 95% confidence interval (CI): 1.019~1.028, *P* < 0.01), SD of SBP (HR = 1.019, 95% CI: 1.012~1.026, *P* < 0.01), mean PP (HR = 1.009, 95% CI: 1.002~1.016, *P* < 0.05), SD (HR = 1.020, 95% CI: 1.006~1.034, *P* < 0.01), HbA1c (HR = 1.289, 95% CI: 1.257~1.321, *P* < 0.01), higher LDL-C (HR = 1.006, 95% CI: 1.002~1.010, *P* < 0.01), and lower HDL-C (HR = 0.981, 95% CI: 0.976~0.986, *P* < 0.01) were associated with more rapid development of DR (Table 2).

### 3.3. Cox Regression Analysis after Systolic Pressure Stratification

**Figure 1:**
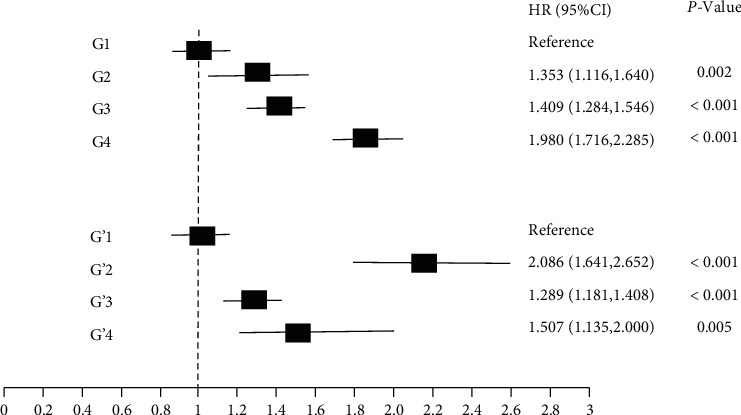
Cox regression on group comparisons. Results were adjusted for age, sex, course of the disease, BMI, waist-to-hip ratio, HbA1c, TC, TG, LDL-C, and HDL-C. G1: mean SBP < 130 mmHg and SD of SBP < 11.16 mmHg; G2: mean SBP < 130 mmHg and SD of SBP ≥ 11.16 mmHg; G3: mean SBP ≥ 130 mmHg and SD of SBP < 11.16 mmHg; G4: mean SBP ≥ 130 mmHg and SD of SBP ≥ 11.16 mmHg. G1′: mean PP < 80 mmHg and SD of PP < 6.53 mmHg; G2′: mean PP < 80 mmHg and SD of PP ≥ 6.53 mmHg; G3′: mean PP ≥ 80 mmHg and SD of PP < 6.53 mmHg; G4′: mean PP ≥ 80 mmHg and SD of PP ≥ 6.53 mmHg.

Cox regression analysis showed that after adjusting for age, sex, course of the disease, BMI, waist-to-hip ratio, HbA1c, TC, TG, LDL-C, and HDL-C, patients in the G4 group had the highest risk of DR (HR = 1.980, 95% CI: 1.716~2.285, *P* < 0.01) and patients in the G1 group had the lowest risk. Also, patients in the G3 group (HR = 1.409, 95% CI: 1.284~1.546, *P* < 0.01) had a higher risk of DR compared to those in the G2 group (HR = 1.353, 95% CI: 1.116~1.640, *P* < 0.01, Figure 1).

### 3.4. Cox Regression Analysis after PP Stratification

Based on a mean PP of 80 mmHg and a SD of the PP of 6.53 mmHg, the patients were regrouped into four groups designated G1′-G4′: G1′ (mean PP < 80 mmHg, SD of PP < 6.53 mmHg), G2′ (mean PP < 80 mmHg, SD ≥ 6.53 mmHg), G3′ (mean PP ≥ 80 mmHg, SD of PP < 6.53 mmHg), and G4′ (mean PP ≥ 130 mmHg, SD of PP ≥ 6.53 mmHg). After adjusting for age, sex, disease course, BMI, waist-to-hip ratio, HbA1c, TC, TG, LDL-C, and HDL-C, Cox regression analysis showed that patients in the G2′ group had the highest risk of DR (HR = 2.086, 95% CI: 1.641~2.652, *P* < 0.01) and patients in the G1′ group had the lowest risk. Also, patients in the G4′ group (HR = 1.507, 95% CI: 1.135~2.000, *P* < 0.01) had a higher risk of DR compared to those in the G3′ group (HR = 1.289, 95% CI: 1.181~1.408, *P* < 0.01, Figure 1).

### 3.5. The Relationship between Changes in Blood Pressure and DR

**Figure 2:**
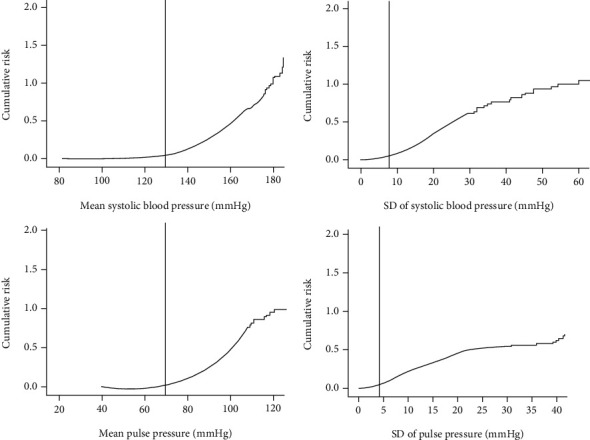
The relationship between systolic blood pressure, pulse pressure, their variability, and diabetes retinopathy.

Compared to the other three groups, patients in the G4 group had the highest risk of DR. The risk of DR increased between our study groups when the mean systolic pressure exceeded 130 mmHg, the SD of SBP exceeded 8 mmHg, the mean PP exceeded 70 mmHg, and the SD of PP exceeded 4 mmHg (Figure 2).

## 4. Discussion

### 4.1. Variability in SBP and PP Are Risk Factors for DR in T2DM Patients

Studies have shown [14, 15] that an increase in BPV is independent of blood pressure and can aggravate damage to the target organs of hypertension. Therefore, reducing BPV and maintaining a stable blood pressure are as important as lowering blood pressure. The role of BPV in microvascular disease in diabetes patients has also attracted major attention. However, previous studies [16, 17] have been mostly cross-sectional surveys that did not clarify the causal relationship between variability in SBP, pulse pressure, and DR. This study found that variability in SBP and PP were risk factors for DR in patients with T2DM. Foo et al. [12] showed that a higher mean SBP, but not SBP variability, was significantly correlated with the occurrence of DR in patients with T2DM. This is not consistent with our study. There are several possible reasons for the discrepancy. In comparison to our study, Foo et al. used a smaller sample size (398 cases, compared to the 3275 cases in our study) and had a relatively short follow-up time (average of 2 years compared to an average of 7 years). Also, the duration of diabetes (average 10 years versus an average of 20 years in our study) was relatively short. In addition, 70% of the study population was from Eastern Asia and China, with the remaining 30% from Southeast Asia, Malaysia, and India. In our study, the entire population was from Eastern Asia and China. Another study [18] showed that SBP variability was an independent risk factor for T2DM nephropathy but had no effect on DR. These findings may be due to the small sample used (664 cases versus 3275 cases), the short duration of diabetes (average 5 years versus average 20 years), and a low overall mean SBP SD (9.72 mmHg versus 11.16 mmHg).

Although it is currently unclear how SBP and PP affect DR in T2DM patients, it can be hypothesized that increased blood pressure may damage retinal capillary endothelial cells [19]. Studies of retinal physiology have shown that blood pressure has a role in the pathological changes of DR and participates in the local renin-angiotensin system [20]. Controlling blood pressure can avoid hyperperfusion to reduce the possibility of blood vessel shear injury caused by hypertension. Therefore, reducing the damage of high perfusion to the endothelial cells, blood vessels, and surrounding tissues may help to prevent DR.

Diastolic blood pressure is an important blood pressure parameter. In this study, no correlation was found between diastolic blood pressure and DR in T2DM patients which is consistent with the results from Kawasaki et al. [21] and Rudnisky et al. [22]. This may be because the diastolic blood pressure is more reflective of peripheral vascular resistance and so the arterial function is small, whilst the SBP mainly reflects the hemodynamics of the central aorta. Concerning the physiological and pathological mechanism of DR, endothelial dysfunction tends to cause vasoconstriction rather than vasodilation, and changes in low-resistance arteries may be more important compared to large arterial dysfunction.

The variability in SBP and PP is more harmful to DR than the average SBP and pulse pressure. In clinical practice, physicians should avoid drastically lowering the blood pressure of patients over a short time. This is particularly important in China where the insurance strategy for inpatients is better than that for outpatients. Many hypertensive patients prefer to be hospitalized. Reducing the blood pressure from a relatively high level to a near-normal level within an average time of seven days of hospitalization will inevitably lead to an increase in BPV. These changes affect the occurrence of chronic microvascular complications such as DR. Our data support the development of individualized blood pressure reduction programs to smoothly lower blood pressure and reduce BPV.

### 4.2. The Advantages and Limitations of This Study

Our study has several advantages in that it was performed as a large prospective longitudinal cohort study with a sufficient sample size. Each patient was followed up for at least three years with an average follow-up time of seven years. Also, at least three independent systolic and PP measurements were used to calculate the mean systolic and pulse pressures, as well as the variability in the systolic and pulse pressures. These data provided reliable parameters for the mean systolic and PP as well as the variability of systolic and pulse pressure. One of the main limitations of this study was that during the follow-up period, the number and intervals of systolic and PP measurements varied for each subject per year even though they were supposed to be measured every quarter. Also, as our data were only collected from patients with DR, we are not able to determine if the DR was caused by hypertension or by diabetes. No further analysis of the data was performed to focus specifically on hypertensive DR.

In conclusion, variability in SBP and PP are risk factors for DR in patients with T2DM. This variability may be more important than the mean SBP and PP in the development of DR. In the future, individualized blood pressure reduction programs should be considered in clinical practice to slowly lower blood pressure and improve BPV, thereby delaying the risk of chronic complications of diabetes such as DR.

## Figures and Tables

**Table 1:** Comparison of the general information between the diabetic retinopathy and the nondiabetic retinopathy groups.

Indicators	DR group (*n* = 442)	NDR group (*n* = 2833)	*P* value
Female (number of cases (%))	239 (54.1)	1425 (50.3)	0.607
Age (years, $\bar{x}\pm s$)	67.5 ± 11.3	65.2 ± 12.4	<0.001
BMI (kg/m^2^, $\;\bar{x}\pm s$)	25.9 ± 4.0	26.2 ± 4.2	0.140
Waist-to-hip ratio ($\bar{x}\pm s$)	0.9 ± 0.10	0.9 ± 0.3	0.938
Diabetes course (years, $\bar{x}\pm s$)	23.2 ± 9.5	16.7 ± 7.6	<0.001
Smoking (cases (%))	126 (28.9)	741 (28.6)	0.473
Drinking (cases (%))	97 (22.2)	623 (24.1)	0.224
Exercise (cases (%))	245 (56.2)	1568 (60.6)	0.048
Macular edema (cases (%))	13 (3.0)	12 (0.5)	<0.001
Cataract (cases (%))	231 (53.0)	695 (26.8)	<0.001
Medication			
Hypoglycemic drugs (cases (%))	352 (80.7)	2 166 (83.7)	<0.001
Insulin (cases (%))	104 (23.9)	726 (28.0)	<0.001
Lipid-lowering drugs (cases (%))	294 (67.4)	1772 (68.4)	0.410
Antihypertensive drugs (cases (%))	196 (45.0)	1183 (45.7)	0.544
HbA_1c_ (%)	8.8 ± 2.0	8.1 ± 1.9	<0.001
Systolic pressure (mmHg, $\bar{x}\pm s$)	138.7 ± 15.7	134.5 ± 16.8	<0.001
Diastolic pressure (mmHg, $\bar{x}\pm s$)	78.2 ± 9.2	77.8 ± 9.7	0.462
Pulse pressure (mmHg, $\bar{x}\pm s$)	82.4 ± 11.1	80.3 ± 12.1	0.001
MAP (mmHg, $\bar{x}\pm s$)	101.7 ± 15.3	100.1 ± 16.6	0.062
TC (mg/dl,$\bar{x}\pm s$)	191.5 ± 41.5	188.9 ± 37.3	0.456
TG (mg/dl, median (upper and lower quartile))	126.00 (87.3, 173.0)	124.0 (88.0, 176.3)	0.645
LDL-C (mg/dl, $\bar{x}\pm s$)	109.1 ± 34.2	104.7 ± 30.8	0.074
HDL-C (mg/dl, $\bar{x}\pm s$)	47.6 ± 12.1	48.5 ± 13.5	0.035

Note: DR: diabetic retinopathy; NDR: nondiabetic retinopathy; BMI: body mass index; HbA_1c_: glycosylated hemoglobin; MAP: mean arterial pressure; TC: total cholesterol; TG: triglycerides; LDL-C: low-density lipoprotein cholesterol; HDL-C: high-density lipoprotein cholesterol. ^a^*t* value; ^b^*Z* value.

**Table 2:** Cox regression analysis of risk factors for diabetic retinopathy.

Variables	HR	95% CI	*P* value
Mean systolic blood pressure	1.023	1.019~1.028	<0.001
SD of systolic blood pressure	1.019	1.012~1.026	<0.001
Mean diastolic blood pressure	0.986	0.968~1.005	0.151
SD of diastolic blood pressure	0.999	0.994~1.004	0.601
Mean pulse pressure	1.009	1.002~1.016	0.018
SD of pulse pressure	1.020	1.006~1.034	<0.001
Mean MAP	0.992	0.976~1.009	0.381
SD of MAP	0.996	0.983~1.008	0.496
HbA_1c_	1.289	1.257~1.321	<0.001
TC	0.999	0.996~1.003	0.701
TG	0.999	0.999~1.000	0.059
HDL-C	0.981	0.976~0.986	<0.001
LDL-C	1.006	1.002~1.010	<0.001

Note: MAP: mean arterial pressure; HbA1c: glycosylated hemoglobin; TC: total cholesterol; TG: triglycerides; HDL-C: high-density lipoprotein cholesterol; LDL-C: low-density lipoprotein cholesterol; HR: risk ratio; CI: confidence interval. Correction for age, gender, and disease course. HRs were for per 1-unit increase of each continuous variable.

## Data Availability

The data used to support the findings of this study are available from the corresponding author upon request.
